# Characterization of a SARS-CoV-2 spike protein reference material

**DOI:** 10.1007/s00216-022-04000-y

**Published:** 2022-03-09

**Authors:** Bradley B. Stocks, Marie-Pier Thibeault, Joseph D. Schrag, Jeremy E. Melanson

**Affiliations:** 1 Metrology, National Research Council Canada, 1200 Montreal Road, Ottawa, ON K1A 0R6 Canada; 2 Human Health Therapeutics, National Research Council Canada, 6100 Royalmount Ave, Montreal, QC H4P 2R2 Canada

**Keywords:** COVID-19, SARS-CoV-2, Spike protein, Reference materials, Isotope dilution

## Abstract

Development of diagnostic testing capability has advanced with unprecedented pace in response to the COVID-19 pandemic. An undesirable effect of such speed is a lack of standardization, often leading to unreliable test results. To assist the research community surmount this challenge, the National Research Council Canada has prepared a SARS-CoV-2 spike protein reference material, SMT1-1, as a buffered solution. Value assignment was achieved by amino acid analysis (AAA) by double isotope dilution liquid chromatography–tandem mass spectrometry (LC-ID-MS/MS) following acid hydrolysis of the protein, in combination with ultraviolet–visible spectrophotometry (UV–Vis) based on tryptophan and tyrosine absorbance at 280 nm. Homogeneity of the material was established through spectrophotometric absorbance readings at 280 nm. Transportation and long-term storage stabilities were assessed by monitoring relative changes in oligomeric state by size-exclusion liquid chromatography (LC-SEC) with UV detection. The molar concentration of the spike protein in SMT1-1 was 5.68 ± 0.22 µmol L^−1^ (*k* = 2, 95% CI), with the native trimeric form accounting for ~ 94% of the relative abundance. Reference mass concentration and mass fraction values were calculated using the protein molecular weight and density of the SMT1-1 solution. The spike protein is highly glycosylated which leads to analyte ambiguity when reporting the more commonly used mass concentration. After glycoprotein molar mass determination by LC-SEC with multi-angle light scattering detection, we thus reported mass concentration values for both the protein-only portion and intact glycoprotein as 0.813 ± 0.030 and 1.050 ± 0.068 mg mL^−1^ (*k* = 2), respectively.

**Supplementary Information:**

The online version contains supplementary material available at 10.1007/s00216-022-04000-y.

## Introduction

The recently emerged SARS-CoV-2 coronavirus is the causative agent of the current COVID-19 pandemic which has resulted in nearly 300 million infections and over five million deaths at the time of this report [1]. SARS-CoV-2 infects human airway epithelial cells via binding to the cell-surface receptor angiotensin converting enzyme 2 [2]. The pathogen-host interaction is mediated by the viral spike protein, a multi-domain protein extensively decorated with complex glycans [3]. Neutralizing antibodies (Abs) recovered from plasma of convalescent COVID-19 patients, as well as therapeutic Abs, target various spike protein regions in addition to the attached sugars [4–7]. This multitude of binding modes illustrates the complexity of measuring the spike antibody-antigen interaction, which is the basis for many of the immunoassays currently available commercially.

Public health officials require reliable testing data to inform COVID-19 public policy. While nucleic acid-based diagnostics have been the gold standard, they are resource-intensive, requiring specialized equipment and trained personnel. To facilitate more extensive rapid testing capabilities, multiple point-of-care assays, namely antigen and antibody (serology) tests, have been developed. SARS-CoV-2 antigen tests strive to diagnose current infections by detecting the presence of viral proteins, i.e., spike, in patient samples with immobilized Abs. Conversely, antibody tests utilize immobilized viral proteins to capture Abs from patient serum resulting from a past infection and thus could be employed in viral surveillance programs. Serological assays benefit from ease-of-use and rapid sample turnaround time. However, due to the speed with which these tests have been developed and deployed, rigorous assessment of their accuracy is lacking [8] and the exact antigen/Ab structure often remains undisclosed which raises questions of comparability among manufacturers.

Protein reference materials offer significant advantages through supporting measurement accuracy and reliability of antigen and antibody test kits. Unlike small organic molecules, such as pharmaceuticals, with well-defined structures that can be accurately measured at high precision and accuracy, proteins typically require a range of advanced characterization techniques [9] that can yield larger uncertainties. While matrix reference materials of proteins of interest in serum or nasal fluid would be ideal for validation of test kits, purified proteins in solutions can be valuable as primary calibrators and can be spiked into appropriate matrices for simulating real samples [10].

The recent development of a SARS-CoV-2 nucleic acid reference material allows for enhanced reliability of molecular diagnostics [11], yet to date, protein reference materials are lacking. To address this current lack of standardization in COVID-19 rapid testing, we have developed a SARS-CoV-2 spike protein reference material, SMT1-1 [12]. This material is envisaged to serve as a standardized antigen source for antibody assays as well as a positive control for antigen tests. The production and characterization of this protein reference material will dramatically increase confidence in SARS-CoV-2 immunological testing results.

## Experimental

### Chemicals and standards

NRC CRMs ALEU-1 (leucine), APHE-1 (phenylalanine), and APRO-1 (proline) were used as primary standards for AAA [13]. The amino acid CRMs were assigned purity by quantitative ^1^H-NMR traceable to NIST potassium hydrogen phthalate (SRM 84L). Isoleucine and valine were purchased from Sigma (Oakville, ON). The corresponding isotope-labelled amino acids were sourced from Cambridge Isotope Laboratories (CIL, Tewksbury, MA): [^13^C_6_]-leucine, [ring-^13^C_6_]-phenylalanine, [^13^C_5_]-proline, [^13^C_6_]-isoleucine, and [^13^C_5_]-valine. NIST recombinant humanized IgG1κ monoclonal antibody reference material (NISTmAb, RM 8671) was purchased from NIST (Gaithersburg, MD). Amino acid analysis grade 6 M hydrochloric acid was obtained from Sigma. Acetonitrile, ammonium acetate, phosphate buffered saline, and Tween-20 were purchased from Fisher Scientific (Nepean, ON). Glacial acetic acid was from ACP Chemicals (Montreal, QC) and HEPES sodium salt was purchased from Bioshop (Burlington, ON).

### Preparation of SMT1-1

SARS-CoV-2 spike protein ectodomain mutant trimer was expressed and purified as described previously [14, 15]. The construct included a mutated S1/S2 furin cleavage site and the double proline substitution determined to maintain coronavirus spike proteins in the prefusion conformation [16, 17]. The trimerization domain of human resistin was fused to the C-terminus of the spike protein, followed by FLAG and hexahistidine affinity tags (Fig. S1). Mass spectrometry-based peptide mapping experiments on the deglycosylated protein resulted in > 94% sequence coverage (Fig. S2). The protein was received frozen in formulation buffer (DPBS supplemented with 2% HEPES sodium salt, pH 8). After thawing in a 25 °C water bath, the protein solution was dispensed in 1.2 mL aliquots into sterile 2 mL cryovials, labelled, and stored at − 80 °C.

### Ultraviolet–visible spectrophotometry

Protein concentration in SMT1-1 was determined using a NanoDrop One^c^ (ThermoFisher Scientific, San Jose, CA) UV–Visible spectrophotometer at 280 nm. Instrument performance was verified through analysis of ThermoFisher PV-1 solution, bovine serum albumin standard (Pierce), and NISTmAb. The instrument was blanked with SMT1-1 formulation buffer. Two µL SMT1-1 solution was pipetted onto the pedestal and the pathlength was set to 0.1 cm. Protein concentration was determined via the Beer-Lambert equation with an extinction coefficient of 140 320 M^−1^ cm^−1^, predicted from the spike amino acid sequence with 18 disulfide bonds [18].

### Size-exclusion chromatography

Liquid chromatography was performed on a Waters Acquity UPLC (Milford, MA) equipped with a BEHSEC 450 Å, 4.6 × 150 mm column, thermostatted at 30 °C. The autosampler was maintained at 4 °C. Ten µL (~ 8 µg) SMT1-1 was injected onto the column and eluted at 0.4 mL min^−1^ with formulation buffer supplemented with 0.02% Tween-20. Solvent was directed into the flow cell of a Waters Acquity eλ photodiode array detector operating at 280 nm with 1.2 nm resolution. Data analysis was done with MassLynx 4.1 software provided by the instrument manufacturer. Column and detector performance were confirmed with Waters BEH450 SEC standard protein mix and NISTmAb RM8671. Multi-angle light scattering (MALS) and refractive index (RI) data were collected using miniDAWN and Optilab T-rEX detectors, respectively (Wyatt, Santa Barbara, CA) and data were analyzed using the accompanying Astra software (version 6.1.7.17).

### Amino acid analysis by LC-ID-MS/MS

Amino acid stock solutions were prepared gravimetrically in ultrapure water. A calibration blend was formulated through combination of natural Ile, Leu, Phe, Pro, and Val stocks, with the final mole fractions mimicking those present in the spike protein sequence. To facilitate exact-matching isotope dilution, a corresponding blend of the ^13^C-labelled amino acids was prepared as internal standard. Equal amounts of the internal standard were then spiked into both the calibration blend and SMT1-1, followed by liquid phase acid hydrolysis in 6 M HCl. The solutions were heated at 110 °C for 72 h to ensure complete peptide bond cleavage [19, 20], then allowed to cool to room temperature. A 40 μL aliquot of each solution was diluted to 1 mL prior to LC–MS/MS analysis. A NISTmAb RM 8671 sample was prepared in parallel as a quality control, after a tenfold dilution of the initial concentrated solution.

Analyte separation was achieved using an Ultimate 3000 UHPLC (ThermoFisher Scientific) employing a ZIC-HILIC column (Canadian Life Science, 100 × 2.1 mm, 3.5 μm) heated to 35 °C. Mobile phases were respectively (A) 10 mM ammonium acetate in water with pH adjusted to 3.5 using glacial acetic acid and (B) 10 mM ammonium acetate in ACN. Two μL injections were used and separated isocratically with 88% B at 0.25 mL min^−1^. Eluting amino acids were directed into the heated electrospray source of a Thermo Quantiva triple quadrupole MS operating in multiple reaction monitoring (MRM) mode. Compound specific parameters were first optimized by infusing individual amino acid standards: ion transitions (Q1/Q3), collision energy (CE), and radio-frequency (RF) values used are shown in Table S1. The ESI spray voltage was set to + 4400 V and the sheath, auxiliary, and sweep gas flows were 25, 17, and 1, respectively (arbitrary units). The ion transfer tube and vaporizer temperatures were set to 325 and 280 °C, respectively. Peak areas were extracted using Xcalibur software from the instrument manufacturer.

Quantitation of each amino acid in the sample (*w*_A_) was achieved using the following double isotope dilution equation [21]:

1 $$w_{\mathrm A}=w_{\mathrm A^\ast}\cdot\frac{r_{\mathrm A^\ast}-r_{\mathrm A^\ast\mathrm B}}{r_{\mathrm A^\ast\mathrm B}-r_{\mathrm B}}\cdot\frac{r_{\mathrm B}-r_{\mathrm{AB}}}{r_{\mathrm{AB}}-r_{\mathrm A^\ast}}\cdot\frac{m_{\mathrm A^\ast\left(\mathrm A^\ast\mathrm B\right)}}{m_{\mathrm B\left(\mathrm A^\ast\mathrm B\right)}}\cdot\frac{m_{\mathrm B\left(\mathrm{AB}\right)}}{m_{\mathrm A\left(\mathrm{AB}\right)}}$$

where A represents the analyte in the sample, A* the primary standard (natural amino acid), B the internal standard (labelled amino acid), A*B the calibration blend, and AB the sample blend. Symbols *w*, *r*, and *m* denote the mass fraction, the measured isotope amount ratio, and the mass of solution, respectively. Validation of this technique has been demonstrated in inter-laboratory comparisons [22, 23], and has facilitated purity assessment of multiple peptide RMs [24–26].

## Results and discussion

### Homogeneity of SMT1-1

**Fig. 1:**
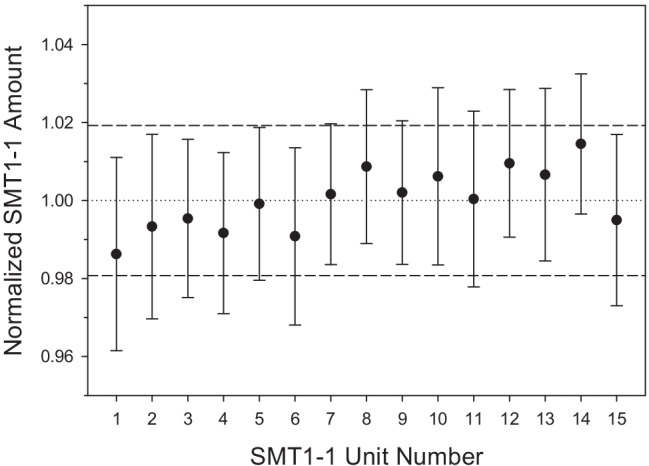
Normalized protein content in fifteen randomly selected SMT1-1 units. Data points and error bars represent averages and standard deviations, respectively, of triplicate technical replicates. Dotted line is the average molar concentration determined by A_280_ and dashed lines indicate standard uncertainty (*k* = 1)

Solution-phase reference materials are often highly homogeneous; however, inhomogeneity can occur throughout the production process for a variety of reasons, i.e., changing environmental conditions, interaction between the sample and its container, and contamination. Therefore, using the molar protein concentration measured by UV–Vis at 280 nm, a homogeneity assessment was performed and a corresponding uncertainty component determined. Fifteen units were randomly selected from the batch, and each unit was analyzed in triplicate (Fig. 1). The between unit variability was determined using the Bayesian analysis of variance (ANOVA) with priors set for the mean, the scale, and the half-Cauchy parameters. The model was fit to data using Monte Carlo method using R package *rjags* and the resulting homogeneity uncertainty (*u*_hom_) with 95% confidence interval was 0.021 μmol L^−1^. Because the final SMT1-1 characterization uncertainty (0.22 μmol L^−1^) was > threefold higher than *u*_hom_, the inhomogeneity of the material was determined to be negligible [27].

**Fig. 2:**
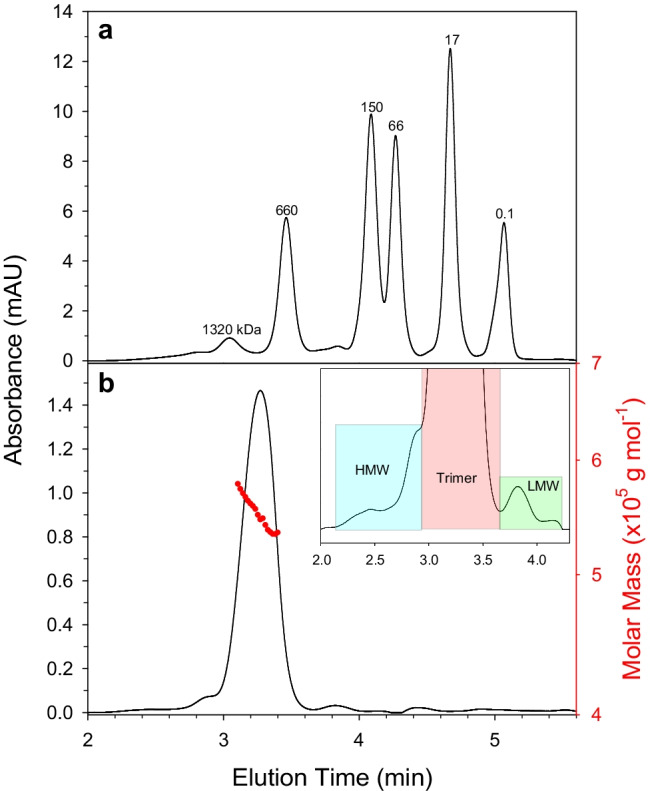
Size-exclusion UV chromatograms of **a** molecular weight standard protein mix, and **b** SMT1-1. Peak labels in **a** indicate molecular weights in kilodaltons. Inset in **b** shows zoom-in on SMT1-1 elution, highlighting regions integrated for the spike protein trimer as well as high- and low-molecular weight species. Molar mass values (red circles, right axis) from MALS detection are overlaid on SMT1-1 peak

A critical factor for protein reference materials is the maintenance of the native higher-order (tertiary, quaternary) structure. The spike protein in SMT1-1 natively forms a non-covalent trimer however these trimers can dissociate or further associate into larger oligomers. The presence of these alternative forms could have effects on downstream assays employing SMT1-1 as a reagent. Therefore, the relative amount of spike trimer in SMT1-1 was assessed by LC-SEC-UV on the same 15 units. Extensive glycosylation causes the spike protein to migrate at nearly double its expected molecular weight by SEC-UV (Fig. 2), however MALS detection revealed that the main peak observed at 3.2 min indeed corresponds to the trimer. The SEC-UV measurements indicated that SMT1-1 consists mostly of trimers (94%) with small amounts of both high and low molecular weight species (4% and 2%, respectively). No significant difference in trimer abundance was detected between units (Fig. S3).

### SMT1-1 stability

**Fig. 3:**
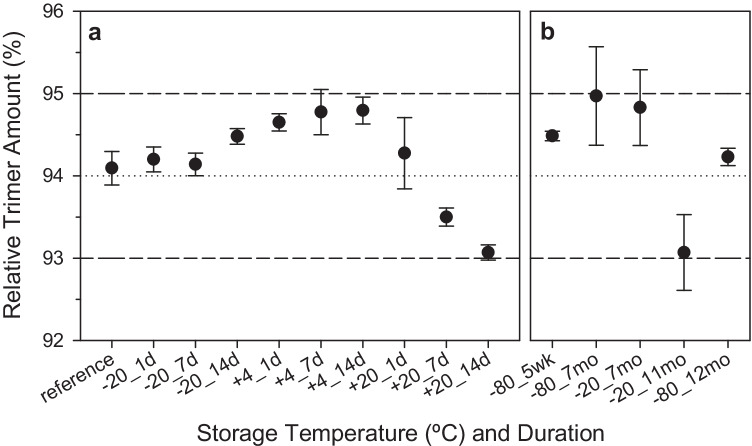
Relative spike trimer amount in SMT1-1 under various storage conditions. Trimer percentage from **a** isochronous short-term stability and **b** long-term stability studies. Reference refers to sample stored at − 80 °C for duration of isochronous study. Values result from triplicate measurements of two units per condition. Data points and error bars represent averages and standard deviations, respectively. The dotted line indicates the average trimer percentage. Dashed lines represent a ± 1% range and are intended for visualization purposes only

As discussed in the preceding section, maintaining the spike protein in its native quaternary structure form is crucial; we therefore assessed the short-term, long-term, and freeze–thaw stability of the trimer in SMT1-1 by LC-SEC-UV. The short-term stability was evaluated to assess potential conditions during transport, as well as during sample preparation and analysis. Using an isochronous approach, duplicate samples were incubated at each of three temperatures (+ 20, + 4, and − 20 °C) for each of three timepoints (1, 7, and 14 days) then analyzed with reference to samples held at − 80 °C. As shown in Fig. 3a, no significant changes in protein size heterogeneity were observed even after 14 days at + 20 °C. However, a small time-dependant decrease in relative trimer amount was apparent upon incubation at + 20 °C, hence extended storage at ambient conditions should be avoided. We again employed Bayesian ANOVA (with half-Cauchy, mean, and scale priors set for the model parameters) along with the Arrhenius equation to link the rate constants at the various temperatures. A short-term stability component was then extracted for 14 days storage at + 20 °C to simulate transportation conditions and at + 4 °C for 1 day to simulate manipulation (sample preparation and analysis) conditions, and no significant difference was found.

The long-term stability of SMT1-1 was determined by supplementing the above data with those from samples stored at − 80 °C for 5 weeks and 7 and 12 months, and at − 20 °C for 7 and 11 months. No significant changes in trimer abundance were detected (Fig. 3b). Extrapolation from the Bayesian ANOVA discussed above supplemented with the long-term data resulted in a stability uncertainty component of 0.04 µmol L^−1^ for 3 years storage at − 20 °C, providing the basis for the anticipated expiry period of the material.

**Fig. 4:**
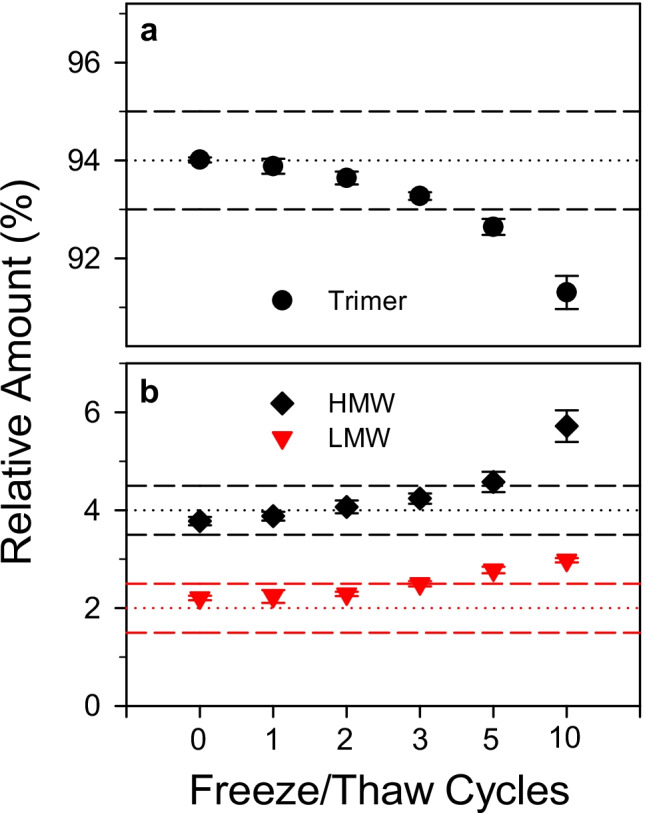
Freeze–thaw effects on protein size heterogeneity in SMT1-1. Relative amounts of **a** trimer and **b** high- and low-molecular weight species as a function of freeze–thaw cycle number. Two SMT1-1 units were used for each condition and analyzed in triplicate. Data points and error bars represent averages and standard deviations, respectively

Finally, SMT1-1 freeze–thaw (F/T) stability was assessed. Duplicate samples were subjected to 1, 2, 3, 5, or 10 F/T cycles, defined as ≥ 1 h incubation at ambient temperature followed by ≥ 2 h storage at − 80 °C. Figure 4 shows that the relative trimer amount decreased as a function of F/T cycle number, with concomitant increases in both the low- and high-molecular weight species. A significant loss in trimer abundance was observed after 5 F/T cycles, therefore subjecting the material to no more than 3 F/T cycles was deemed appropriate.

### Reference value assignment and uncertainty evaluation of SMT1-1

SMT1-1 is a reference material assessed for spike protein content. The reference value reported for spike protein molar concentration combined determinations from two orthogonal methods: UV–Vis spectrophotometry and amino acid analysis ID-MS. Measurements were performed on the same 15 units used for the homogeneity assessment in addition to a NISTmAb quality control sample. Both the UV–Vis ID-MS techniques are sensitive to the presence of host-cell proteins (HCPs), in this case CHO cell proteins co-purified with the spike protein. We were able to detect low-level HCPs in SMT1-1, similar to work on other CHO-based biologics [28]; however, we did not perform detailed quantitation. Therefore, akin to the NISTmAb reference material, we did not include a purity correction for HCPs.

Absorbance readings were recorded on a NanoDrop spectrophotometer. Such microvolume instruments benefit from extremely low (1–2 µL) sample consumption and have been shown to result in < 3% error for amino acid and nucleotide samples [29]. The average concentration of the 15 units was 5.72 ± 0.22 µmol L^−1^ (*k* = 2, 95% CI). The uncertainty incorporates the standard deviations of triplicate measurements, the pathlength error determined during instrument verification, and bias in the concentration determination on the NISTmAb standard resulting from uncertainty in the predicted extinction coefficient. The total protein molar concentration determined by amino acid analysis following protein hydrolysis was 5.64 ± 0.16 µmol L^−1^ (*k* = 2) and the uncertainty results almost entirely from the repeatability of the LC–MS isotope ratio measurements (Fig. S4). The concentrations from the two methods were averaged, and the uncertainties added in quadrature, to give a consensus value of 5.68 ± 0.22 µmol L^−1^ (*k* = 2) (Table 1, Table S2).

**Table 1:** Reference values and expanded uncertainties (*k* = 2, 95% CI) for SMT1-1

Substance	Molar concentration (µmol L^−1^)	Mass fraction (mg g^−1^)	Mass concentration (mg mL^−1^)
SARS-CoV-2 spike protein^a^	5.68 ± 0.22	0.807 ± 0.030	0.813 ± 0.030
SARS-CoV-2 spike glycoprotein^b^	5.68 ± 0.22	1.043 ± 0.068	1.050 ± 0.068

^a^Spike protein amino acid sequence only, 143 192 ± 1 g mol^−1^

^b^Spike protein plus best estimate of total glycan mass, 184 000 ± 10 000 g mol^−1^

Initial SMT1-1 quantitation utilized molar concentrations as these generally do not depend on protein post-translational modifications; absorbance at 280 nm is predominantly mediated by tryptophan and tyrosine side chains. Nevertheless, many protein laboratories routinely work with mass concentrations (mg mL^−1^) rather than molarities which spurred us to also provide such values for SMT1-1. For many proteins this is a simple calculation involving the molecular weight, however the extensive glycosylation of the spike protein introduces ambiguity regarding the analyte in question, as discussed in the ensuing section.

### Molecular weight determination

The SARS-CoV-2 spike protein sequence contains 22 potential N-glycosylation sites per monomer (Fig. S1). MS-based glycoproteomic studies have been applied to determine both the glycan occupancy and composition at each site [30–32], with the total glycan mass per trimer totalling 90–105 kDa. The N-glycosylation profile has been shown to differ dramatically depending on the protein construct employed, i.e., full-length spike, S1 domain, or receptor binding domain [33, 34], and the cell type used for recombinant protein production. Recent charge detection MS measurements of the intact spike trimer indicate a glycan mass ~ 40% higher [35] than that reported in proteomic studies, akin to the discrepancy observed for erythropoietin [36].

Calculating the theoretical molecular weight (MW) of a protein is a straightforward exercise, combining the elemental composition of the amino acid sequence and the corresponding IUPAC atomic weights [37], and accurate experimental MW determination of intact proteins with LC–MS has become routine. Mass spectrometry facilitates detection of well-defined post-translational modifications, such as acetylation and phosphorylation, however the notoriously heterogeneous nature of N-glycosylation significantly increases measurement uncertainty when even a small number of labelling sites are present within the protein sequence. This is apparent through comparison of both the *m/z*- and deconvoluted mass spectra of native and deglycosylated spike (calculated MW = 143 214 ± 1 g mol^−1^) shown in Figure S5.

Size-exclusion chromatography coupled with UV detection was utilized to determine the size heterogeneity of SMT1-1. Calibrants used for SEC are generally globular, non-glycosylated proteins. However, the SARS-CoV-2 spike in SMT1-1 is highly glycosylated and stabilized in an elongated conformation [3, 14, 38]. These factors predictably alter protein SEC elution profiles [39], causing the spike trimer MW to be over-estimated by nearly 100% (Fig. 2b). Additionally, the large peak width in comparison to those of the calibrants reflects the heterogeneous nature of the glycosylation [35]. Recent work utilizing SEC separation coupled with MALS and differential refractive index (dRI) detection was able to accurately determine the MW of the extensively glycosylated HIV-1 envelope trimer [40]. We therefore measured SMT1-1 by SEC-MALS (Fig. 2b) and determined an intensity-weighted trimer molar mass of approximately 551 kDa. This value implies ~ 122 kDa glycan present on the spike trimer in SMT1-1, which falls between the values calculated from glycoproteomics (~ 100 kDa) and charge detection MS studies (~ 135 kDa) [35]. Mass concentration (mg mL^−1^) of the spike glycoprotein monomer in SMT1-1 was thus calculated from the molar concentration using a best estimate molecular mass of 184 000 ± 10 000 g mol^−1^. The ~ 5% uncertainty (*k* = 2) in the molar mass of the spike glycoprotein comes from the standard deviation of replicate SEC-MALS analyses, and reflects the heterogeneity in glycoforms across the SEC elution peak. Mass concentrations were converted to mass fractions using a density value of 1.007 ± 0.008 g mL^−1^, measured on the actual SMT1-1 solution.

## Conclusions

While vaccinations have reduced mortality associated with COVID-19, infection diagnostics and viral surveillance remain critical aspects of the global pandemic response. Rapid testing kits, both antigen- and antibody-based, play a key role in diagnosing current and prior SARS-CoV-2 infections. As a result, over 150 such tests have been authorized for use in the USA and Canada alone [41, 42], however comparability between manufacturers remains unclear. We have characterized a spike protein reference material, SMT1-1, envisaged to be used as a standard protein source for developing SARS-CoV-2 serological and antigen tests. SMT1-1 can also be used as a SI-traceable calibrator in the development of spike protein quantitation assays.

## Supplementary Information

Below is the link to the electronic supplementary material.

Supplementary file1 (DOCX 691 KB)
