# Supplementary material for: Characterization of a SARS-CoV-2 spike protein reference material
Source: Anal Bioanal Chem. 2022 Mar 9;414(12):3561–9. doi: 10.1007/s00216-022-04000-y (PMC8904068; doi:10.1007/s00216-022-04000-y)
Supplement: Supplementary file 1 — Supplementary file1 (DOCX 691 KB) [file 216_2022_4000_MOESM1_ESM.docx]

*Electronic Supplementary Information*

**Characterization of a SARS-CoV-2 Spike Protein Reference Material**

Bradley B. Stocks^1^, Marie-Pier Thibeault^1^, Joseph D. Schrag^2^, and Jeremy E. Melanson^1^

^1^*Metrology, National Research Council Canada*

*1200 Montreal Road, Ottawa, ON, Canada K1A 0R6*

^2^*Human Health Therapeutics, National Research Council Canada*

*6100 Royalmount Ave., Montreal, QC, Canada H4P 2R2*

**TABLE S1**

| **Amino**  **Acid** | **Q1**  **(m/z)** | **Q3**  **(m/z)** | **RF**  **(V)** | **CE**  **(eV)** | **Dwell**  **(msec)** | **RT**  **(min)** |
| --- | --- | --- | --- | --- | --- | --- |
| Phe | 166.1 | 120.2 | 48 | 14 | 50 | 4.40 |
| Phe-^13^C_6_ | 172.1 | 126.2 | 48 | 14 | 50 | 4.40 |
| Leu-Ile | 132.1 | 86.2 | 44 | 12 | 50 | 5.15 – 5.85 |
| Leu-Ile-^13^C_6_ | 138.1 | 91.2 | 44 | 12 | 50 | 5.15 – 5.85 |
| Val | 118.1 | 72.2 | 48 | 11 | 50 | 8.69 |
| Val-^13^C_5_ | 123.1 | 76.2 | 48 | 12 | 50 | 8.69 |
| Pro | 116.1 | 70.2 | 47 | 16 | 50 | 11.22 |
| Pro-^13^C_5_ | 121.1 | 74.2 | 47 | 16 | 50 | 11.22 |

**Table S1.** LC-MS/MS acquisition parameters for amino acid quantitation

**TABLE S2**

| **Substance** | ***U*_k=2_** | ***u*_c_** | ***u*_char_** | ***u*_hom_** | ***u*_stability_** |
| --- | --- | --- | --- | --- | --- |
| SARS-CoV-2 spike glycoprotein molar concentration (µmol/L) | 0.22 | 0.11 | 0.10 | 0.00 | 0.04 |
|  |  |  |  |  |  |
| SARS-CoV-2 spike protein only mass fraction (mg/g) | 0.03 | 0.015 | 0.014 | 0.000 | 0.01 |
|  |  |  |  |  |  |
| SARS-CoV-2 spike glycoprotein mass fraction (mg/g) | 0.068 | 0.034 | 0.033 | 0.000 | 0.01 |
|  |  |  |  |  |  |

**Table S2.** Uncertainty components of reference values for SMT1-1

**FIGURE S1**

**
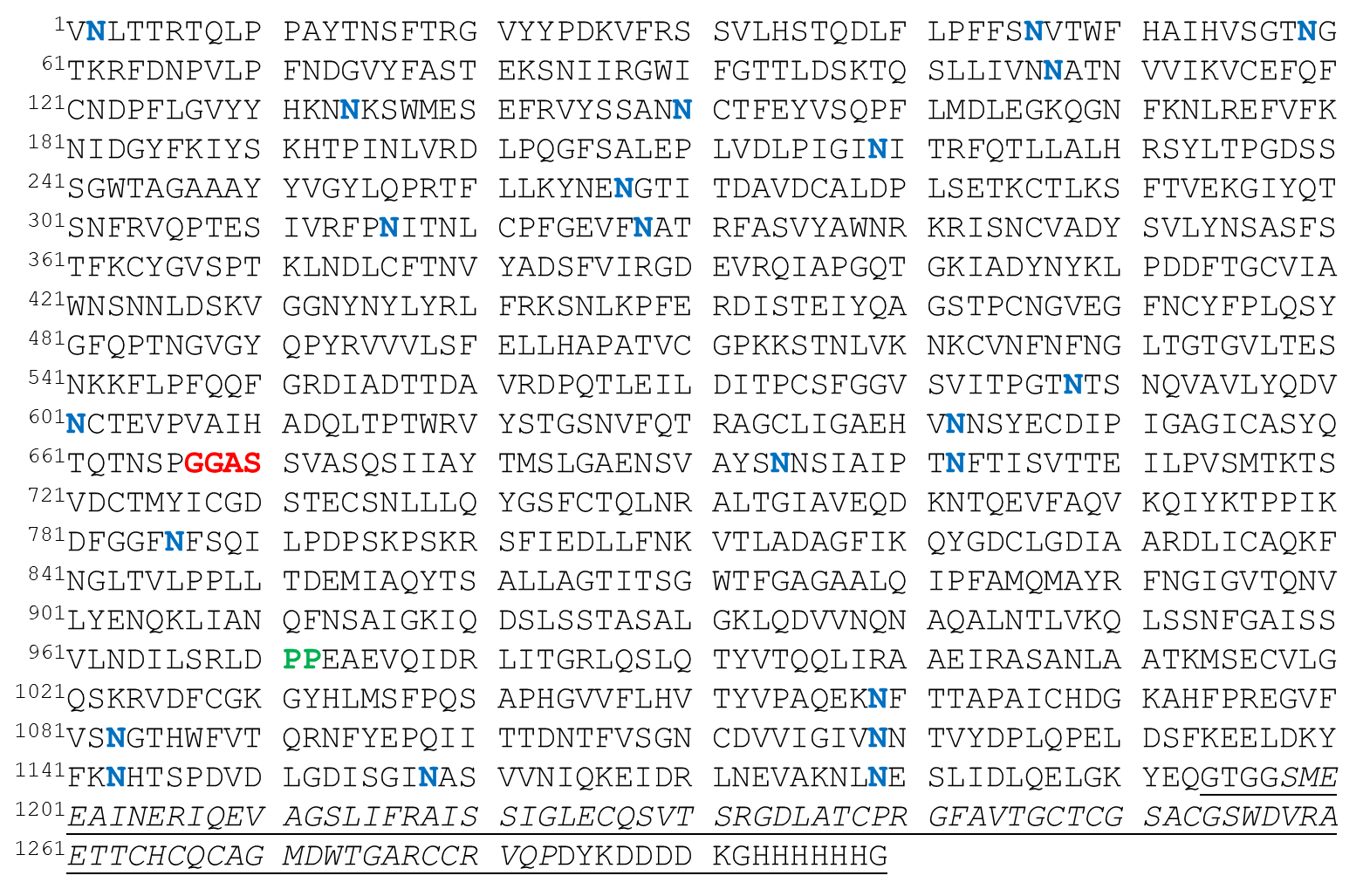
**

**Figure S1.** Amino acid sequence of spike protein construct in SMT1-1. The mutated furin cleavage site is shown in red, stabilizing proline mutations are colored green, and glycosylation sites are shown in blue. SARS-CoV-2 spike protein sequence is depicted in normal text (non-underlined), followed by the trimerization domain from human resistin (italic) and FLAG and 6x-His affinity tags.

**FIGURE S2**

**
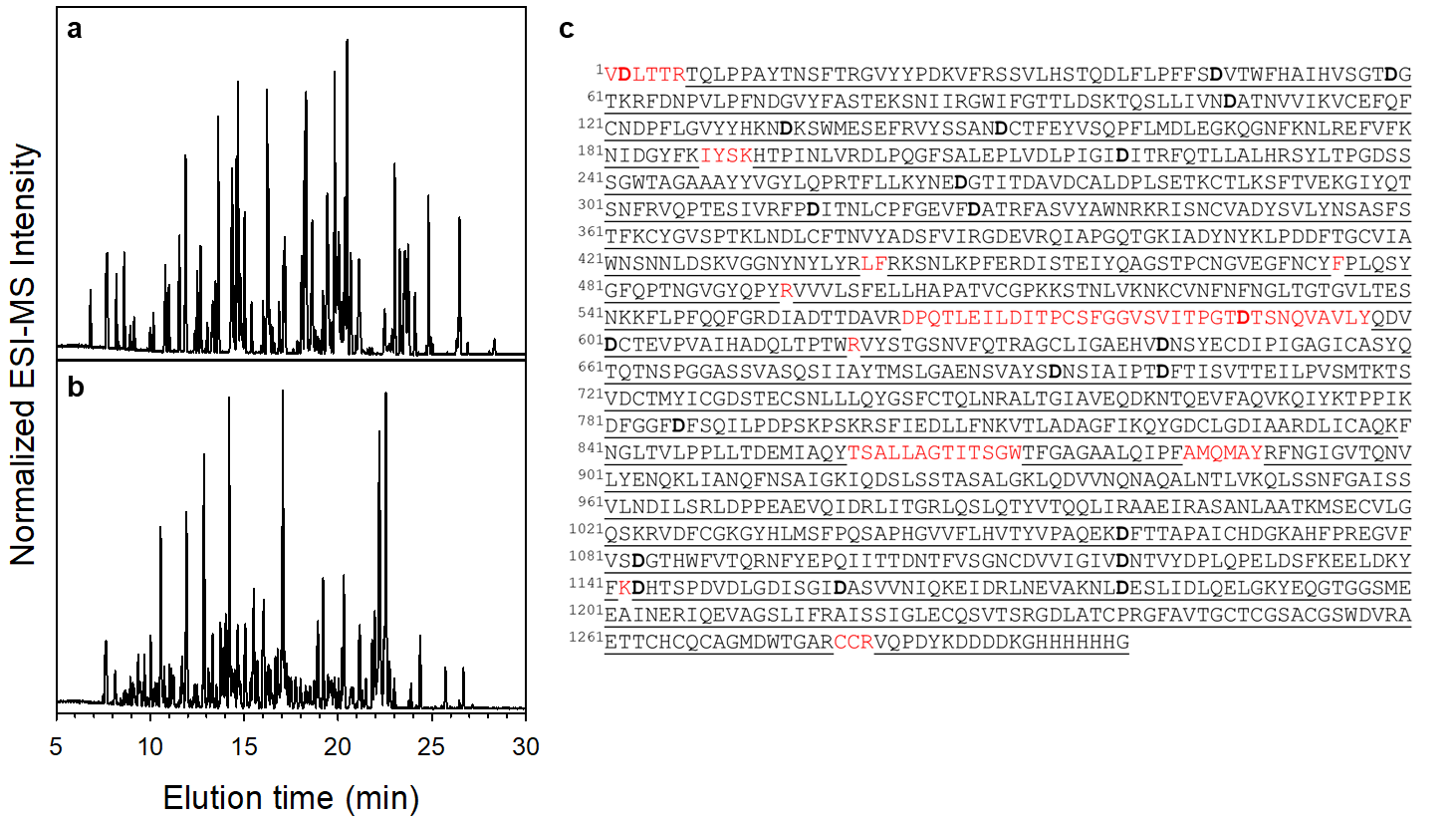
**

**Figure S2.** Deglycosylated spike protein sequence coverage from MS-based peptide mapping. LC-MS chromatograms resulting from (A) trypsin and (B) chymotrypsin digests. Peaks in panel B at 14.2 and 22.5 min have been scaled by 0.5. (C) Underlined sequence corresponds to peptides identified by MS/MS. Peptides not identified are coloured red. Aspartic acid residues shown in bold result from PNGase F deglycosylation of asparagine residues.

**FIGURE S3**

**Figure S3.** Normalized trimer amount in fifteen randomly selected SMT1-1 units. Data points and error bars represent averages and standard deviations, respectively, of triplicate technical replicates. Dotted line is the normalized average trimer percentage determined by LC-SEC-UV.

**FIGURE S4**

**Figure S4.** Normalized protein content in SMT1-1 determined by amino acid analysis ID‑MS after acid hydrolysis. The fifteen units measured are identical to those depicted in Figure 1 of the main text. The spike protein amount was determined from the mole fraction of each of the five amino acids measured (Phe, Leu, Ile, Val, Pro) and their respective abundance in the SMT1 protein sequence. Vertical bars and error bars represent averages and standard deviations, respectively, of triplicate technical replicates. Dotted line is the average protein amount determined by ID-MS and dashed lines indicate standard uncertainty (*k* = 1).

**FIGURE S5**

**
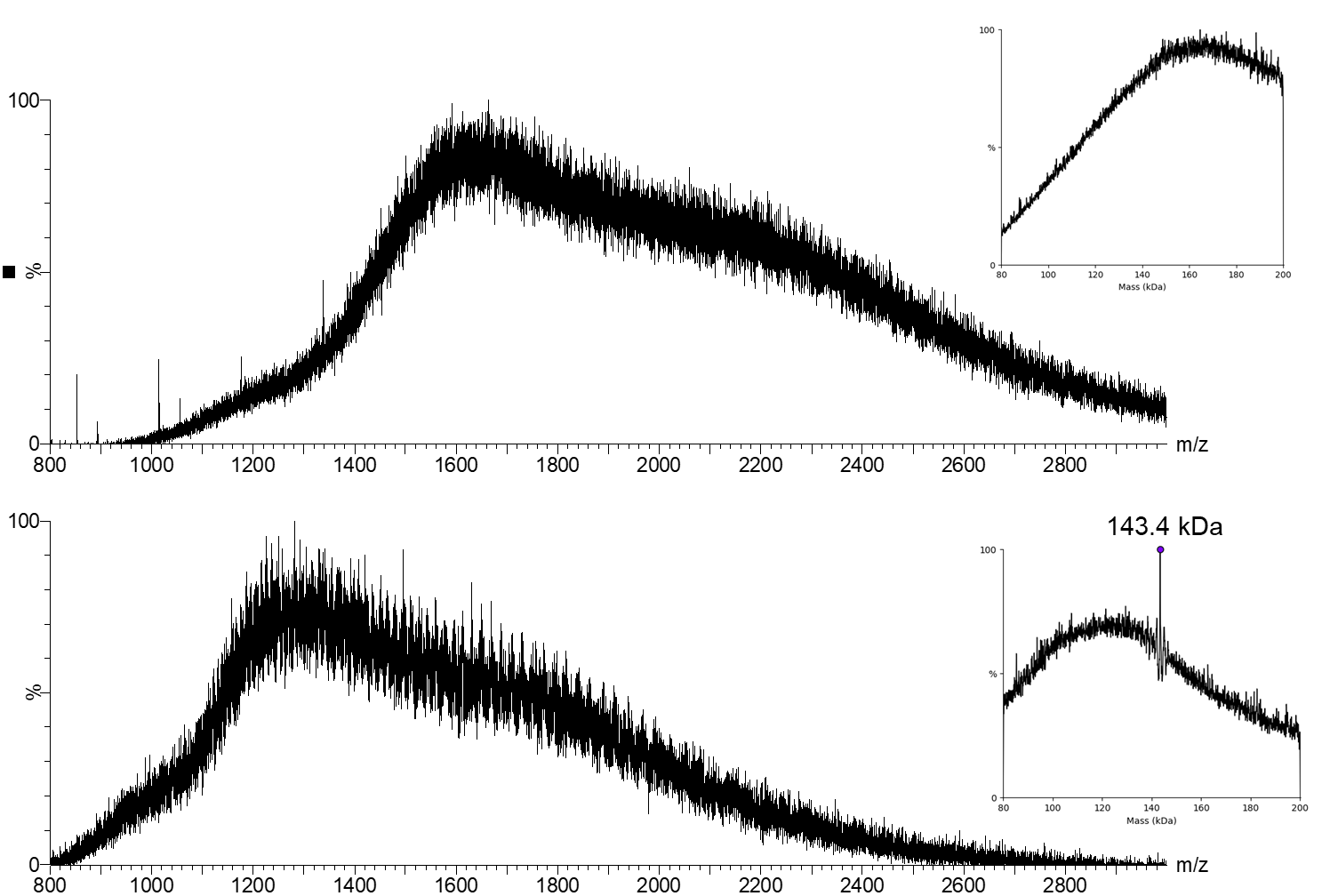
**

**Figure S4.** LC-MS spectra of native (top panel) and deglycosylated (bottom) spike protein. Insets show the corresponding deconvoluted mass spectra generated with UniDec [1].

1. Marty M. T., Baldwin A. J., Marklund E. G., Hochberg G. K., Benesch J. L., Robinson C. V. Bayesian deconvolution of mass and ion mobility spectra: from binary interactions to polydisperse ensembles. Anal Chem. 2015;87:4370-4376. https://doi.org/10.1021/acs.analchem.5b00140.
